# Commentary: Effects of psilocybin on time perception and temporal control of behavior in humans

**DOI:** 10.3389/fpsyg.2016.00736

**Published:** 2016-05-19

**Authors:** Katarina L. Shebloski, James M. Broadway

**Affiliations:** ^1^Department of Psychology, University of California, Santa BarbaraSanta Barbara, CA, USA; ^2^Department of Neuroscience, University of New MexicoAlbuquerque, NM, USA

**Keywords:** subjective time perception, temporal processing, psilocybin, 5-HT2A receptor, schizophrenia, serotonin, altered states of consciousness

## Introduction

We discuss Wittmann et al. (2007) “Effects of Psilocybin on Time Perception and Temporal Control of Behavior in Humans,” proposing that altered states of consciousness induced by pharmacological treatments and neurological disorders can reveal much about the circuitry underlying time perception in normal states of consciousness. Further research is needed to integrate these separate research domains.

The brain integrates partial sensory input with internal representations to construct the elaborate story we know as time (Hammond, 2012). Our ordinary experiences reveal the complicated game the mind can play with perceived time: the day drags when we are bored, yet slips through our fingers when distracted or amused. Despite varying phenomenological experiences of time, the appropriate integration of physical time with functional behavior requires a sufficiently accurate perception structured by the conceptual framework of past, present, and future (Eagleman et al., 2005). Working memory, attention, and executive control support this integrated construction (Fuchs, 2007; Marchetti, 2014). Effects on human time perception are observed when these cognitive systems are modulated by pharmacological treatments or psychiatric disorders (González-Maeso and Sealfon, 2009), suggesting the presence of a neurophysiological process that is intrinsic to temporal information processing (Rammsayer, 2008).

## Psilocybin and temporal processing in normal subjects

Serotonergic hallucinogens generally slow the perceived flow of time (Shanon, 2003). Pharmacological manipulations using psilocybin have shed light on mechanisms responsible for this distorted time experience. Wittmann et al. (2007) investigated time estimation under the influence of psilocybin. The study addressed the functional role of serotoninergic 5-HT_2A_ receptors in internal clock models (ICMs) in duration discrimination and temporal control of motor performance. The study revealed a decreased ability to accurately produce intervals longer than 3 s and synchronize finger-tapping to auditory beats separated by more than 2 s. This suggests that effects of psilocybin on temporal processing are specific to relatively long durations, attributable to memory, and decision-making components of the ICM (Gibbon et al., 1984; Block and Zakay, 1996; Rammsayer, 2008; Allman and Meck, 2012), rather than to more basic pacemaker/accumulator mechanisms (Wittmann et al., 2007).

Comparable results are observed in Wackermann et al. (2008), assessing psilocybin duration reproductions of intervals between 1.5 and 5 s. The analyses rely on the “dual klepsydra” model (DKM), a contemporary alternative to the ICM. In the study, the DKM model is applied to Wittmann et al. (2007) data as well as new data. Results indicate temporal processing influenced by psilocybin are dose-dependent (Wackermann et al., 2008).

Temporal processing of longer durations is impaired in people with schizophrenia (Fuchs, 2005; Bonnot et al., 2011). However, recent meta-analyses suggest that timing deficits in schizophrenia generalize across sub- and supra-second intervals, as well as across perceptual and motor tasks; and are independent from more generalized cognitive impairments (Alústiza et al., 2016; Ciullo et al., 2016). The employment of psychoactive substances may be a useful approach to understanding temporal processing in both the ordinary brain and that which is affected by psychiatric disorders.

## 5-HT_2A_ receptors and temporal processing in schizophrenia

Psychopharmacological research suggests that drugs such as psilocybin may serve as useful tools for understanding temporal serotonergic signaling mechanisms underlying psychosis, due to their capacity to cause distorted perception in normal subjects (Rammsayer, 2008; González-Maeso and Sealfon, 2009). Modulated 5-HT_2A_ receptor agonists may induce clinical symptoms of schizophrenia such as hallucinations, delusion, psychomotor poverty, and distorted perception (Teixeira et al., 2013), including distorted time perception (Allman and Meck, 2012). Pharmaceutical alterations of 5-HT_2A_R activation have shown to assist NMDAR-dependent memory mechanisms (Zhang and Stackman, 2015), and demonstrate that altered time perception is a defining characteristic in schizophrenia due to cognitive changes from NMDA receptor antagonists (Ciullo et al., 2016). Additionally, dopamine-release manipulations cause motor and cognitive defects seen in schizophrenia (Raote et al., 2007), and impair duration discrimination in healthy subjects (Wittmann, 2009). Likewise, schizophrenia is associated with poor accumulation of signal durations derived from impairments in sensory integration (Allman and Meck, 2012; Teixeira et al., 2013). Sysoeva et al. (2010) found that genotypes characterized by higher 5-HT transmission exemplify a higher “loss rate” of duration representation, which may correlate to the very high 5-HT_2A_ R occupancy in the prefrontal cortex of schizophrenic patients (Zhang and Stackman, 2015).

Impairments in working memory, selective attention, and executive control, as seen in schizophrenia, lead to distorted sequencing and integration of past, present, and future into a personal narrative. Carter et al. (2005) demonstrate a reduction in attentional tracking abilities affected by psilocybin, and implicate 5-HT receptors in these processes through pretreatment of the 5-HT_2A_ receptor antagonist ketanserin.

## Proposal for integrative research

5-HT_2A_ receptor activity is associated with time distortion in both psychiatric disorders and hallucinogenic experiences. Manipulating antagonists/agonists provides an approach to utilizing psychoactive drugs as tools in research for understanding time perception in the ordinary brain. It would be fruitful to compare healthy subjects under the influence of psilocybin with patients with acute schizophrenia, utilizing a common paradigm as in Wittmann et al. (2007). However, Wittmann et al. (2007) excludes significant moderating factors of time estimation: attention and emotion (Droit-Volet and Meck, 2007). An fMRI test of acute treatment with psilocybin in healthy volunteers found decreased amygdala reactivity during emotion processing (Kraehenmann et al., 2015). Negative pictures led to an overestimation of duration, indicating greater attention allotted to emotional valence (Wittmann, 2009).

Neuroimaging techniques combined with psychophysical tests of time perception (for a review see Grondin, 2010), including manipulations to assess attentional and emotional factors, will illuminate neural activity responsible for temporal processing in schizophrenia and psychedelic perceptions. Comparing performance and brain activities in these altered states with those of untreated healthy subjects under the same experimental conditions will elucidate mechanisms underlying time perception.

## Conclusion

Slowing of perceived time is induced by psilocybin and schizophrenia; having a common basis in 5-HT_2*A*_ receptor activities. Commonalities across pharmacological treatments and neurological disorders should be explored within a common experimental paradigm to better understand neurochemical processes mediating temporal processing in ordinary states.

## Author contributions

KS obtained comprehensive research and drafted original article, with collaboration of JB's efforts. JB provided substantial advisory of research materials and writing processes. Both authors edited and revised the article, insured the integrity of the product, and agreed on the final version for submission.

### Conflict of interest statement

The authors declare that the research was conducted in the absence of any commercial or financial relationships that could be construed as a potential conflict of interest.
